# Autoimmune/Autoinflammatory Syndrome Induced by Adjuvants (ASIA Syndrome) After Polypropylene Mesh Implantation – Protocol of a Pilot Study for Diagnostics and Treatment

**DOI:** 10.3389/jaws.2025.14266

**Published:** 2025-06-26

**Authors:** M. J. C. A. M. Gielen, K. L. C. van Rest, N. D. Bouvy, G. A. van Koeveringe, C. R. Kowalik, J. P. W. R. Roovers, R. M. H. Roumen, M. C. Slot, H. P. J. Willems, W. A. R. Zwaans

**Affiliations:** ^1^ GROW Research Institute for Oncology and Reproduction, Maastricht University, Maastricht, Netherlands; ^2^ Department of Surgery, Máxima Medical Centre, Eindhoven, Netherlands; ^3^ Department of Surgery, Maastricht University Medical Centre, Maastricht, Netherlands; ^4^ Department of Obstetrics and Gynaecology, Amsterdam University Medical Centres, Amsterdam, Netherlands; ^5^ Amsterdam Reproduction and Development Research Institute, Amsterdam, Netherlands; ^6^ Department of Urology, Maastricht University Medical Centre, Maastricht, Netherlands; ^7^ Bergman Clinics, Amsterdam, Netherlands; ^8^ Research Consortium Mesh, Utrecht, Netherlands; ^9^ SolviMáx Centre of Excellence for Abdominal Wall and Groin Pain, Eindhoven, Netherlands; ^10^ Department of Allergology and Clinical Immunology, Maastricht University Medical Centre, Maastricht, Netherlands; ^11^ Department of Internal Medicine, Máxima Medical Centre, Eindhoven, Netherlands; ^12^ NUTRIM School of Nutrition and Translational Research in Metabolism, Maastricht University, Maastricht, Netherlands

**Keywords:** ASIA syndrome, polypropylene, mesh complications, implant reaction, systemic autoimmune disorders

## Abstract

**Background:**

An increasingly vocal movement of patients with systemic complaints, supposedly linked to polypropylene mesh implants, is leading to increasing numbers of patient-preferred mesh-less surgical repairs for inguinal hernia, stress urinary incontinence, and pelvic organ prolapse. However, current literature does not support any association between polypropylene implants and Autoimmune Syndrome Induced by Adjuvants (ASIA). This prospective pilot aims to examine autoimmunity in patients in whom ASIA is suspected, based on previously described criteria. We aim to demonstrate the effectiveness of mesh allergy testing and to investigate the natural evolvement of ASIA symptoms or the effect of mesh removal on ASIA complaints.

**Methods:**

This multi-centre, prospective pilot study will include patients with symptoms of the ASIA syndrome according to Shoenfeld’s Criteria. Physical examination, immunologic blood tests, and mesh allergy tests will be performed by an experienced immunologist and surgeon. Questionnaires on improvement of symptoms, psychological susceptibility, and connective tissue disease will be collected at predefined time points. When patients’ wish for mesh removal is persistent, mesh implants will be removed surgically. All meshes will be assessed histopathologically. Follow-up is 12 months.

**Discussion:**

Current evidence on a causal relation between polypropylene mesh and ASIA syndrome is lacking. In this study, mesh allergy testing will be evaluated as a potential first objective screening test for inflammation-like response specific to polypropylene, in patients meeting diagnostic criteria for ASIA syndrome following polypropylene implantation. This study will be performed to add to existing literature on ASIA with polypropylene adjuvants and to help reduce knowledge gaps on diagnosis and prognosis.

## Introduction

Inguinal hernia repair is one of the most frequently performed surgeries in the Netherlands (27,000 to 30,000 yearly), with a vast variety in possible approaches. Whereas initial surgical procedures had significant risk of recurrence, the introduction of polypropylene (PP) meshes reduced the incidence of recurrence drastically [1–3]. Nowadays, a mesh-based repair is the preferential treatment, either endoscopically or via an ‘open’ approach [4, 5]. Subsequently, synthetic mesh has found its way into other pelvic reconstructive surgery. Stress Urinary Incontinence (SUI) and Pelvic Organ Prolapse (POP) have been treated with PP mid urethral slings and mesh implants respectively, since the ‘90s. The rationale for the use of PP-implants is similar; the high recurrence rate (14%–17%) after autologous repair declined significantly after introduction of the use of mesh [4, 6, 7].

As recurrence risk has greatly declined in abdominal wall and urogynaecological surgery, research attention has shifted towards other complications. Approximately 0.5%–6% of patients operated for inguinal hernia develop chronic postoperative inguinal pain (CPIP) that severely influences daily life and limits work performance [4]. In POP or SUI repair, mesh erosion and mesh exposure can lead to extensive morbidity, ultimately discouraging the use of mesh implants in these procedures [7, 8]. Apart from CPIP, and mesh erosion/exposure in vaginal PP mesh implantation, patients seldomly report subjective systemic complaints after polypropylene mesh implantation, such as chronic fatigue, pyrexia, and myalgia [9, 10].

However, in the last three odd years these non-specific, potentially mesh-related, complaints after pelvic and inguinal hernia repair have gained awareness in the (inter)national scientific society whereas several media have highlighted these refractory postoperative complications. In research and lawsuits, patients’ concerns are rapidly increasing in suit. Due to earlier safety problems with transvaginal meshes and growing concerns of the possibility of a chronic foreign body reaction to the mesh, patients now unconditionally oppose polypropylene mesh implants [11]. Concurrently, hernia mesh lawsuits are becoming more prevalent in the United States as well as in the Netherlands [12–14].

Local morbidity associated with the implantation of mesh in urogynaecological surgery has led to a more negative connotation of mesh in these fields. While not directly attributable to its increasing popularity, widespread media coverage on the negative effects of implants in recent decades, such as breast implant illness, seems to correlate strongly with the increased interest of patients and their advocates in polypropylene-related illness [15–18]. For instance, some patients report inflammation-like symptoms following polypropylene mesh implantation, suggesting the presence of a systemic autoimmune response or a so called “Autoimmune/autoinflammatory Syndrome Induced by Adjuvants” (ASIA syndrome, formerly known as Shoenfeld’s syndrome) [19, 20]. These symptoms include myalgia, arthralgia, chronic fatigue, fever, swelling and other non-specific and subjective symptoms.

Despite differences in composition of these implants, their placement in the human body and the proposed mechanism(s) of induction of illness (such as silicone migration or encapsulation), form the rationale for the grouping of all implants and implant-related complications to an autoimmune disorder or syndrome [21]. Concerns regarding these effects of various implants occupy the minds of both professionals and patients [16, 21]. Subsequently, data on true incidence of autoimmune diseases after polypropylene implantation are currently unavailable. However, estimations are up to 3%, which is equal to its incidence in the general population [6, 22].

So far, solid evidence of a causal relation between mesh implantation and systemic autoimmune or autoinflammatory response is lacking. In 2021, a systematic review and meta-analysis was performed considering all available evidence on the association between PP implants for pelvic floor surgery and inguinal hernia repair and autoimmune/autoinflammatory diseases [23]. It was concluded that: *“to date, there is no evidence to suggest a causal relationship between implantation of PP mesh and the occurrence of autoimmune disease”* [23].

In the present pilot study, a possible relation between the implantation of PP mesh for inguinal hernia repair, SUI or POP and subsequent systemic autoimmune complaints will be investigated by testing immunologic and allergic responses before and, on indication, after mesh removal. Additional value of Mesh Allergy Testing (MAT) shall be assessed as a measure to objectify these complaints. If so, a profound insight in diagnostics for systematic complaints will be attained that may provide new directions for future treatment.

### Study Registration

This study is registered under NCT06363903 (clinicaltrials.gov). Current stage: Enrolment.

## Methods

### Study Design

In this multicentre pilot cohort study, five departments in three Dutch centres will be participating: department of General Surgery and department of Allergology, Máxima Medical Centre (Máxima MC, Veldhoven), department of Gynaecology, Amsterdam University Medical Centre and department of Urology and department of Immunology, Maastricht University Medical Centre (MUMC+; Maastricht). Each department will have an outpatient clinic for potential ASIA patients after PP implantation. Patient follow-up will be 1 year. Patients expressing a persisting wish for mesh removal may undergo complete mesh removal if deemed safe by the surgeon. If intra-operative findings lead to limitations in complete removal, mesh will be partially removed. Patients not undergoing mesh removal will be assessed for spontaneous relief of symptoms. These patients can be used as naturalistic comparison against the group of patients that eventually undergo mesh explantation.

### Participants

Fifty consecutive patients with suspected systemic autoimmune/autoinflammatory response or ASIA syndrome will be recruited from one of the five aforementioned departments. In the present study, patients are considered to have ASIA when at least three major criteria from Table 1 are present, as previously published [17]. Only referrals from the outpatient ASIA clinics will be accepted and no active recruitment will be undertaken. Subjects will be eligible for inclusion if they meet all of the following criteria:

- Patients >18 years of age and written informed consent obtained;
- Suspected ASIA syndrome after elective inguinal hernia, POP, or SUI repair, defined as at least three major ASIA criteria (Table 1), of which the exposure to an external stimulus (PP mesh) is one;
- ASIA complaints started specifically after the implantation of PP mesh and were not present before the surgical repair.

**TABLE 1:** Diagnostic criteria autoimmune/autoinflammatory syndrome induced by adjuvants (ASIA) syndrome [17].

Major criteria
- Removal of inciting agent induces improvement
- Exposure to an external stimulus (infection, vaccine, silicone, adjuvant) prior to clinical manifestations
- The appearance of ‘typical’ clinical manifestations:
- Myalgia, myositis or muscle weakness
- Arthralgia and/or arthritis
- Chronic fatigue, unrefreshing sleep or sleep disturbances
- Neurological manifestations (especially associated with demyelination)
- Cognitive impairment, memory loss
- Pyrexia
- Sicca (dry mouth and/or dry eyes)
- Typical biopsy of involved organs
Minor criteria
- The appearance of autoantibodies or antibodies directed at the suspected adjuvant
- Other clinical manifestations (e.g., irritable bowel syndrome)
- Specific HLA (e.g., HLA DRB1, HLA DQB1)
- Evolvement of an autoimmune disease (e.g., multiple sclerosis, systemic sclerosis)

Patients will be excluded from participation in this study if one or more of the following is present:

- Known autoimmune/inflammatory disorders;
- Known malignancies;
- (Low grade) infections or other inflammatory diseases at time of surgery;
- Cognitively impaired individuals.
- Recent (<3 months) vaccination

Sample size calculation is not applicable as the present study is a pilot and preceding literature about the validity of MAT is absent, but considering the low expected number of cases annually, with strict inclusion criteria (at least 3 major ASIA criteria present), and no active recruitment in only three centres, total number of proposed inclusions has been estimated restrictively, in order not to misjudge availability of inclusions.

### Interventions

After inclusion, patients will be asked to fill out several questionnaires and to visit the immunology outpatient clinic (T0) to undergo a MAT and blood testing. For the MAT, 1 mL methyl ethyl ketone (MEK) is used to dissolve a 5 cm^2^ mesh for at least 48h, to max 72 h. 25 μL of the MEK solution and part of the mesh are tested topically in a Finn chamber. MAT is considered positive when a dermal reaction on the topical applicant occurs within 48 h, as assessed by an immunologist in Máxima MC or MUMC+.

At first visit, blood samples from all subjects will be obtained as standard diagnostic immunologic tests in which the following will be determined: C-reactive protein (CRP), haemoglobin, erythrocyte sedimentation rate (ESR), leukocytes, differentiation of leukocytes (including eosinophil count), thrombocyte count, creatinine, creatine kinase (CK), albumin, alanine transaminase (ALT), ANA, anti-dsDNA, ANCA, Rheumatoid factor (Rf), anti-CCP, and if indicated total level of protein, interleukin-6 (IL-6), total IgG and subclasses. Additionally, an extra blood sample will be obtained for diagnostic purposes to exclude specific systemic or autoimmune diseases if any of the determinations is abnormal.

Patients will be asked to fill out a set of questionnaires to assess subjective complaints, changes of complaints over time and changes related to mesh removal (if applicable). The Patient Global Impression of Improvement (PGI-I), Pain Catastrophizing Scale (PCS), Hospital Anxiety and Depression Scale (HADS), Short Form Health Survey (SF-12), and the Connective Tissue Disease Screening Questionnaire (CSQ) will be obtained. Patients are asked to complete these at T0, T1 and T2. In case of mesh removal, T1 and T2 will be respectively 6 and 12 months after mesh removal. All patients, independent of MAT and blood sample outcomes or spontaneous resolution of systemic complaints, will be followed up in the first year after inclusion.

### Outcomes

Primary outcome is the value of MAT as an objective diagnostic test for patients who fit the current ASIA criteria. Secondary outcomes include the value of immunologic blood tests, and the association between both subjective outcomes (autoimmune/autoinflammatory symptoms) and objective findings (MAT, blood test).

Another secondary outcome is the success rate of (partial) mesh removal for the resolution of subjective complaints. This success rate is defined as a complete resolution of subjective complaints (on CSQ) and ASIA symptoms (Table 1) and the report of a “much better” or “very much better” condition on PGI-I.

Baseline and dynamic patient demographics will be collected, including age, gender, ASA classification (American Society of Anaesthesiologists), year of PP implantation and type of PP implant, duration of ASIA complaints, pain scores (using Numerical Rating Scale; NRS), body mass index (BMI) smoking status, medication use (including immunosuppressants), previous imaging, time of resolution of symptoms, and remaining symptoms.

Additionally, in case of surgical mesh removal, the following parameters will be collected: intraoperative macroscopic configuration of mesh, duration of surgery, additionally performed neurectomy, and complications. All explanted meshes will be examined histopathologically to check for granulomatous inflammatory or acute inflammatory reactions surrounding the explant.

### Statistical Methods

Baseline patient demographics will be presented as descriptive statistics. Primary outcome (rate of positive diagnostic tests) will be presented as descriptive statistics (percentage of positive tests) at all predefined time points.

Secondary outcomes include the association between subjective (questionnaire outcomes evaluating systemic symptoms or ASIA symptoms) and objective findings (MAT, blood test). For this outcome, subjective complaints are summed (each complaint is 1 point). Objective findings are dichotomized. The association between the number of subjective complaints and the presence of objective findings is subsequently calculated via Chi-Square Test or Fisher’s Exact Test in low expected counts, where applicable. For missing data, percentage of data present will be described. If known, a rationale for missing data will be presented. If only limited data is missing per variable, analysis will proceed as expected. Imputation of missing data is not preferred as imputation within the relatively small sample size would decrease validity of the study, as well as the intention to present real data as it is.

A p-value <0.05 is considered statistically significant. Success rates of (partial) mesh removal will be presented as descriptive statistics (percentage of successful surgery).

Patients are considered to have ASIA when either two major or one major and two minor criteria are present (adapted from Cohen Tervaert 2018) [17]. In the present study, we only considered patients to have ASIA when at least three major criteria are present.

## Discussion

To our knowledge, this study is the first to date to prospectively evaluate the difficulties and unknowns in pragmatic diagnosis, natural progression, and quality of life of ASIA-afflicted patients after PP implantation. Due to concerns within society regarding PP implants being a safety hazard, physicians are left with an unresolved problem as scientific literature is scarce. Therefore, it is likely to result in unnecessary diagnostics and high healthcare costs without any proven benefit to the patient. Prospective studies on the causal relation between adjuvants and ASIA are limited, and lacking regarding PP. Furthermore, medical treatment is currently targeted unequivocally at completely removing the adjuvant, or reducing a generalized immune response, with proof of its effectiveness consisting predominantly of selected case reports and case series [23–25]. This pilot is the first step to systematically examine patients with possible PP-associated ASIA and their complaints in relation to time, objective blood and screening tests, (effectiveness of) operative treatment, and self-reported complaints via validated questionnaires. Without this evidence, caution is warranted in supporting unproved and potentially unsafe treatments leading to redundant or excessive (re-)operations [26–28].

A series of studies have been published on the relationship between adjuvants that can cause inflammation and their possible relation to autoimmune diseases or ASIA. Most of these studies offer either a hypothetical pathophysiological basis for symptoms [19, 29], or describe a series of vastly varying cases with heterogeneous triggers for the immune response [17, 20, 30]. Systematically obtained proof of correlation between PP implants and ASIA remains to be published [31]. Kowalik et al. (2021) performed a systematic review and found little literature on an association between PP and ASIA [23]. Furthermore, they found no evidence of a correlation between PP and systemic autoimmune syndromes [23]. A second systematic review, performed by Jisova et al. (2023) found that multiple studies show no clear evidence linking PP implantation to ASIA. Studies reporting evidence of autoimmunity after mesh implantation were accompanied by high risk of bias, insufficient reporting of confounders, and absence of relevant distinction between autoinflammation and chronic foreign body reaction. Both reviews included the retrospective cohort study of Chughtai et al. (2017), which investigated the onset of autoimmune diseases after interventions. The paper included colonoscopy patients as a control group and used exact individual matching for comparison [22]. Chughtai found no difference in incidence of autoimmune disease between the colonoscopy group (1.6%; n = 25,432) and PP group (1.5%; n = 12,716) after an average of 6 years follow-up [22]. In the present pilot study, patients are prospectively included and are comprehensively tested during the study period. Patients who are not included in the group that undergo surgical removal of the prosthesis could function as a naturalistic control group to those patients that received surgical removal. Considering included patients undergo similar exposure, blood sample collection and follow-up data retrieval, this might lead to potential future analytical strategies. We aspire to provide stronger evidence to base future correlational analysis on and subsequently lead to evidence-based practice for physicians that treat these patients.

This pilot aims to assess the diagnostic value of Mesh Allergy Testing as a first objective screening tool, to aid physicians in decision-making for possible treatments (including high-risk mesh removal) [26, 27]. MAT has not been validated as a test for screening systemic reaction to an adjuvant before, necessitating the current exploratory nature of this study. While findings in physical examination and medical history can aid in a differential diagnosis, testing patients for susceptible HLA-types or antibodies related to mesh has unknown predictive values. Currently, only subjective patient or observer described diagnostic modalities are considered in the ASIA criteria, leading to a lack of specificity in discerning patients with ASIA from other syndromes (Table 1) [17]. The current pilot therefore intentionally opposes earlier case series on ASIA by only including patients with at least three major ASIA criteria, to form a narrower but more specific patient population. Consequently, to assess the value of MAT, patients who suffer from light ASIA symptoms or who only slightly match the diagnosis, will be excluded to create a homogeneous population of patients who fit the criteria of ASIA most optimally, to test value of MAT [32]. In addition, patients will be referred to an immunologist to test for identifiable autoimmune diseases and relevant confounders, and to exclude other illnesses that could correctly explain the presented symptoms.

In our pilot, the explanted meshes will all be sent for histopathologic examination to identify ongoing microscopic inflammatory processes and to relate these to potentially positive MAT results, despite earlier research by Fadaee et al. (2019) showing minor to no benefits of this test as proof of any relation between PP and ASIA [33]. Additionally, while presence of (chronic) inflammation, foreign body reaction, and fibrosis surrounding the mesh explant are to be expected, reports of pathology findings can lead to a deeper understanding of the difference between autoimmunity and (normal) foreign body reactions in patients suffering from ASIA [33]. Objective diagnostic modalities for ASIA are essential, and a rapid, low-cost, non-invasive diagnostic test such as MAT could be a vast improvement in the diagnosis of patients; if results are promising and consistent [32].

Whilst the existence of ASIA has not yet been solidified, it is vital that physicians take patients presenting with these complaints seriously [34, 35], as other mechanisms may (also) underlay these complaints. A stubborn fear amongst patients exists that medical specialists receive funding from professional organizations and industries, to hide the true damage of suggested treatments [12, 13, 36–39]. This is particularly true for patients who have developed complaints after PP mesh implantation as a considerable number visit numerous specialists and charlatans with expensive and ineffective treatments [11]. The misunderstanding of physicians may lead to fear, and sometimes anger aimed at the surgeon who implanted the PP prosthesis. This makes it difficult to construct a trustful bond between patient and physician, although essential for optimal treatment results. For these patients it is essential for the physician to undertake effort to grow trust, to abstain from expressing any derogative comments, and to level with the patient and their insecurities. As the earned trust is fickle and delicate, taking a comprehensive anamnesis is vital and emphasis should be put on the complaints of the patient and their impact. The onset of symptoms in relation to the PP implantation, and presence of pain, may give further clues for diagnosis. When signs of immune activation are present, it is highly recommended to rule out other known autoimmune disorders. Surgeons should be hesitant to ‘blindly’ perform surgical removal of the implant, considering risks for necessary follow-up surgery [28, 40]. Considering the large number of PP implants placed globally every year, it is striking that there is still so little literature on the existence of ASIA after PP implantation [41]. Until research yields higher quality evidence of the causal relationship between PP implants and autoimmunity, future perspectives for patients suffering from ASIA are grim, as misinformation, misunderstanding, and misguidance of patients will remain.

### Limitations

MAT is used to test for type IV allergic reactions as a possible underlying mechanism for systemic complaints. There is no literature regarding this mechanism so far, and the effectiveness and meaning of the MAT for ASIA patients is uncertain. The proportion of patients that present with all symptoms supporting presence of ASIA as stated by Shoenfeld’s criteria, but with a negative MAT is yet to be deduced [17]. Furthermore, as this pilot is not a clinical trial and treatment is not randomized, it is likely that patients with complaints will opt for surgical removal of the PP implant, making it increasingly difficult to illustrate the normal progression of ASIA symptoms. Note should be taken that the effectivity of surgical removal is not the primary aim of this pilot! The natural course of complaints of ASIA patients will be displayed for future studies to further base new standards of care on.

## Conclusion

As the use of PP implants for hernia, SUI and POP repair is successful in the vast majority of patients; surgeons, (uro)gynaecologists and urologists wish to continue these mesh-based techniques to have optimal results and to prevent recurrences. Taking available literature into consideration, the incidence of PP-based ASIA symptoms is probably very low. No causal relation between PP mesh and development of autoimmune syndromes has been demonstrated to date, and considering the substantial risk of co-morbidity of surgical mesh removal, surgeons should be hesitant to operate these patients for this indication. Until the present study has been carried out, physicians should be cautious to suggest that PP implants cause autoimmune syndromes. These suggestions can distress patients, shifting patients’ preference to operative interventions without proper indication and potentially harm them. Furthermore, patients who experience ASIA symptoms and attribute these to their mesh placement, will often seek treatments that are not (yet) supported by evidence. This could lead to more harm than benefit when not meeting their predefined expectations, increasing their distrust in healthcare in general [42, 43]. Instead, physicians should discuss with these patients that the available evidence does not indicate a causal association between PP implants and autoimmune syndromes. When patients present with symptoms indicating excessive immune activation, the treating physician should exclude other autoimmune diseases before applying the ASIA diagnostic criteria. Currently, patients suffering from ASIA-like complaints are doomed to a lack of understanding from their peers and scarce information and treatment from professionals, due to insubstantial evidence that corroborates any association between polypropylene mesh implants and their complaints. The present study will aim towards new evidence-based insights for diagnosis and treatment of these patients.

## Data Availability

The raw data supporting the conclusions of this article will be made available by the authors, without undue reservation.

## Abbreviations

ALT, Alanine Transaminase; AMC, Amsterdam Medical Centre; ANA, Antinuclear Antibodies; ANCA, Antineutrophilic Cytoplasmic Antibody; Anti-CCP, Anti-cyclic Citrullinated Peptide; Anti-dsDNA, Anti-double stranded DNA; ASA, American Society of Anaesthesiologists; ASIA, Autoimmune Syndrome Induced by Adjuvants; BMI, Body Mass Index; CK, Creatine Kinase; CPIP, Chronic Postoperative Inguinal Pain; CRP, C-reactive protein; CSQ, Connective Tissue Disease Screening Questionnaire; ESR, Erythrocyte Sedimentation Rate; HADS, Hospital Anxiety and Depression Scale; HLA, Human Leukocyte Antigen; IgG, Immunoglobulin G; IL-6, Interleukin-6; MAT, Mesh Allergy Testing; MC, Medical Centre; MEK, Methyl Ethyl Ketone; MUMC+, Maastricht University Medical Centre; NRS, Numerical Rating Scale; PCS, Pain Catastrophizing Scale; PGI-I, Patient Global Impression of Improvement; POP, Pelvic Organ Prolapse; PP, polypropylene; RF, Rheumatoid Factor; SF-12, Short Form Health Survey; SUI, Stress Urinary Incontinence.
